# Establishment of a genome map-based karyotype of *Artemisia argyi* and identification of a new octoploid

**DOI:** 10.3389/fpls.2025.1621415

**Published:** 2025-06-25

**Authors:** Lina Li, Pei Du, Dahui Liu, Xiangyang Li, Guixiao La, Guixia Shi, Dandan Dai, Tiegang Yang

**Affiliations:** ^1^ Provincial Key Laboratory of Conservation and Utilization of Traditional Chinese Medicine Resources, Institute of Chinese Herbal Medicines, Henan Academy of Agricultural Sciences, Zhengzhou, Henan, China; ^2^ College of Pharmacy, Hubei University of Chinese Medicine, Wuhan, China

**Keywords:** A*rtemisia argyi*, repetitive oligonucleotide, karyotype, octoploid, moxa

## Abstract

*Artemisia argyi*, an essential plant in traditional Chinese medicine, encounters significant challenges in the development of germplasm resources and cytological research. This study employed the *A. argyi* reference genome to develop 20 repetitive sequence oligonucleotide (oligo) probes, all of which produced clear signals on the chromosomes of the cultivar Qicun Xiang Ai (QCXA). These probes were configured into two probe cocktails (Multiplex #1 and Multiplex #2) that effectively generated chromosome signals under non-denaturing hybridization conditions through probe staining. By integrating probe staining with 45S rDNA fluorescence *in situ* hybridization (FISH) and electronic localization techniques, we established a genome map-based karyotype for QCXA that corresponded to its genomic sequence map. Utilizing this karyotype, we identified almost all chromosomes of the cultivars Wan Ai Ls-9 (WALs-9), Anguo Qi Ai (AGQA), and Anyang Bei Ai (AYBA) and investigated meiotic chromosome pairing behavior in WALs-9. These findings suggest that *A. argyi* may be a distinctive allotetraploid with a base chromosome number of 17. while AYBA (x = 8) appears to be a related species. Furthermore, a novel octoploid germplasm (APLs-9) is successfully generated and characterized through chromosomal doubling, demonstrating significantly enhanced moxa length and moxa content per leaf area - traits with substantial potential for improving both quality and yield. The developed octoploid and high-resolution karyotyping system are poised to significantly advance *A. argyi* breeding and production.

## 1 Introduction

*Artemisia argyi*, a perennial *Asteraceae* species with global distribution, finds extensively application in herbal medicine, cosmetics, fragrances, and other industries (Wei et al., 2024). While China dominates global *A. argyi* production, with Nanyang “Wan Ai” representing the largest cultivation area, progress in yield and quality improvement remains constrained by limited germplasm resources and systematic breeding approaches. The plant’s medicinal value primarily derives from leaf extracts (moxa and *Artemisia* oil) renowned for their therapeutic effects against dampness, bleeding, and inflammation (Wang et al., 2006).

Polyploid breeding, involving chromosome doubling to enhance desirable traits, shows particular promise for vegetatively propagated species like *A. argyi*. This approach typically yields plants with thicker leaves, darker pigmentation, enlarged organs. The increase in gene dosage also leads to significant increases in the levels of certain nutrients or secondary metabolites (Vyas et al., 2007; Abdoli et al., 2013; Thong-On et al., 2014; Wang et al., 2021), yet remains unexplored for *A. argyi* improvement.

The genus *Artemisia* exhibits two primary chromosomal base-number classifications: species with x=8 (ranging from diploid to hexaploid) and those with x=9 (spanning diploid to dodecaploid) (McArthur and Sanderson, 1999; Torrell and Vallès, 2001). However, ploidy levels within *Artemisia* demonstrated considerable variation. Recent genetic studies have identified *A. argyi* is an allopolyploid plant possessing 34 chromosomes, including two remarkably elongated chromosomes (Miao et al., 2022). Gene collinearity analysis indicates these elongated chromosomes represent fusion products of ancestral chromosomes 8 and 9, resulting in *A. argyi* unique tetraploid constitution with a base number of 17 (Miao et al., 2022). This complex chromosomal architecture poses significant challenges for conventional cytogenetic analysis, particularly in elucidating genomic structure and genetic composition.

Seven repetitive sequence single-stranded oligonucleotide (SSON) probes developed using unassembled genome reads of *A. annua*, *A. vulgaris*, and *A. viridisquama* can generate banding signals on *A. argyi* chromosomes (He et al., 2024), but diverse cytological markers specific to this species are still needed. This limitation hinders deeper insights into genetic diversity, chromosomal structural variations, and genomic classification. Eukaryotic genomes contain abundant repetitive DNA sequences that maintain relative intraspecies stability while showing substantial interspecies divergence. These sequences serve as valuable tools for chromosome or genome identification (Fu et al., 2015; Du et al., 2023), karyotype analyses (Du et al., 2018), detection of structural chromosomal variations (Fu et al., 2021), genome differentiation assessments (Braz et al., 2018; Alioto et al., 2020), and identification of distant hybrid progeny (Xi et al., 2020). Oligo fluorescence *in situ* hybridization (Oligo-FISH) employing single-copy or repetitive sequences has proven particularly effective for high-resolution karyotyping in crops, including maize, wheat, and peanuts (Zhuang et al., 2015; Du et al., 2017; Zhu et al., 2017). The recent chromosome-level assembly of *A. argyi* cultivar “Xiang Ai” (8.03 Gb) through PacBio-Hifi sequencing and Hi-C technology (Miao et al., 2022). The aims of the present study were twofold: (1) development of *A. argyi*-specific oligo probes for establishing high-resolution karyotypes, and (2) chromosomal doubling of “Wan Ai” to generate novel breeding germplasm.

## 2 Materials and methods

### 2.1 Plant materials

The experimental materials comprised three cultivars of *A. argyi*, namely, Qichun ‘Xiang Ai’ (QCXA), Wan Ai Ls-9 (WALs-9), and Anguo Qi Ai (AGQA), and the *A. vulgaris* cultivar Anyang Bei Ai (AYBA). Detailed information regarding germplasm sources, accession numbers, and genomic data is provided in **Supplementary Table S1**.

### 2.2 Chromosome preparation

All materials were cultured at 26°C. Root tips of tissue culture seedlings, once they reached 1-1.5 cm in lengths, were excised and placed in an ice–water mixture for 24 h. Subsequently, the root tips were then fixed in 3v:1v absolute ethanol: glacial acetic acid at -20°C for three days. Next, Cells were dissociated by treated with 45% acetic acid at room temperature for 1 min before placing on microscope slides for chromosome squashing and observation. After dispersed, the slides were frozen at -80°C for 12 h. Finally, the cover slips were removed to facilitate dehydration of the chromosomes.

### 2.3 Design of oligo probes

The complete genome assembly sequences of QCXA were obtained from NCBI (https://www.ncbi.nlm.nih.gov/datasets/genome/GCA_030686995.1/). The genomes of *A. argyi* were analyzed to identify tandem repeat (TR) sequences using the Tandem Repeats Finder (TRF, version 4.09) with the following parameters: Match = 2; Mismatch = 7; Indel = 7; Probability of match = 80; Probability of indel = 10; Minimum score = 50; and Maximum period = 2000. Overlapping TRs were eliminated utilizing the process_trf_dat2get_best.pl script. The TRs were then filtered using an in-house Python script, with the following parameters: period size ≥10, copy number ≥50, and percent matches >70. Consensus sequences for each cluster were retained using CD-HIT. Consensus monomer sequences with an identity greater than 75% were used to design oligos ranging in length from 40 to 45 nt based on the TRs using Oligo 7 software.

### 2.4 FISH and probe staining

All oligos were modified at their 5′-ends with TAMRA or FAM by General Biosystems Company (Anhui, China) for FISH applications. Plasmids containing wheat 45S rDNA were generously provided by Nanjing Agricultural University. Probes were labeled using fluorescein-12-dUTP (Roche) through the nick translation method. The FISH procedures adhered to those described by Du et al. (2018).

To establish the karyotype of *A. argyi*, two multiplex probe cocktails were developed: Multiplex #1 consisted of TAMRA-modified probes C8-21, C4-13, C10-9, Co-516, C10-7, C5-2, and Tel-1, while Multiplex #2 contained the FAM-modified probes C1-1, C1-12, C10-10, and C5-14. Probe staining was performed according to Du et al. (2019). The probe dye mixture consisted of 40 mL of 2× PBS and 5 µL each of Multiplex #1 (1 µg/µl), Multiplex #2 (1 µg/µl), and DAPI (100 µg/mL). Chromosomes were initially stained with the probe dye for 6 h using the probe dye. After imaging, the chromosomes were washed to remove all signals and dried. Then, the FISH procedure was conducted using the 45S rDNA probe.

### 2.5 Capture of images and analysis

FISH images were captured using a Leica DM6000 fluorescence microscope, equipped with a cooled CCD camera (Leica). Images were optimized for contrast and brightness using Adobe Photoshop. For karyotype analysis, approximately 3 to 5 cells from each cultivar were observed and measured to calculate the average chromosome length and the chromosome arm ratio. Karyotypes were primarily constructed from single cells, unless overlapping chromosomes necessitated composite imaging.

### 2.6 Probe mapping

BLAST searches were performed against the *A. argyi* reference genome using oligo probes C8-21, C4-13, C10-9, Co-516, C10-7, C5-2, Tel-1, C1-1, C1-12, C10-10, and C5-14 as well as wheat 45S rDNA plasmid probes (http://mcgb.uestc.edu.cn/b2dsc). The number and positions of sequences exhibiting homology greater than 80% in *A. argyi* were recorded. Chromosome positions with either >30 matches for any probe combination or > 1 match per megabase for the wheat 45S rDNA plasmid were mapped using Mapchart.

### 2.7 Meiotic analysis

Young tassels containing anthers at appropriate meiotic stages were harvested and fixed in fixative solution (3v:1v ethanol: acetic acid) at room temperature for 24 h, Then stored at −20°C until further use. One anther from each flower was examined to select pollen mother cells (PMCs) at the diakinesis of meiotic phase I, while the remaining anthers were pressed to prepare chromosome slides.

### 2.8 Chromosome doubling and pollen fertility analysis

The chromosome doubling method employed in this study was based on the interspecific hybrid F_1_ doubling technique for peanut (Li et al., 2017; Wang et al., 2021). In brief, under tissue culture conditions, healthy WALs-9 seedlings were excised from the lower stem below the third leaf from the apex. The apical shoot segment was placed on solid MS medium supplemented with 0.01 g/L NAA, 3% (w/v) sucrose, and 0.05% (w/v) colchicine. The culture was maintained for 8–10 days under 16 h of light and 8 h of dim light daily. Once leaf yellowing and curling were observed, the seedlings were transferred to solid MS medium supplemented with 0.01 g/L NAA and 3% sucrose for recovery, until plants with fully developed roots, stems, and leaves were established.

The doubled plants were subsequently transplanted into the field, with non-doubled plants serving as controls. During the full bloom period, flowers were collected, placed on slides, and crushed using forceps. One to two drops of aceto-carmine solution were added before cover slips were positioned atop them. Pollen fertility was assessed under a microscope by counting ten flowers with three fields of view per flower. Pollen grains that appeared regular in shape and stained red were classified as fertile, whereas irregular or unstained pollen grains were deemed sterile. Pollen fertility was calculated using the following formula: Pollen fertility = (Number of fertile pollen grains)/(Number of fertile pollen grains + Number of sterile pollen grains) × 100%.

### 2.9 Phenotypic investigation

Prior to harvest, comprehensive phenotypic evaluations of WALs-9 and the chromosome doubled *A. argyi* plants were conducted, focusing on moxa length and content quantification. Plant height measurements were obtained from five randomly selected plants per group. For determination, twenty leaves were collected from identical node positions. Moxa collection was performed by carefully harvesting material from leaf undersurfaces using fine tweezers, ensuring inclusion of adjacent parenchyma cells. Thirty biological replicates were mounted on microscope slides (secured with black tape) for stereomicroscopic measurement the length and documentation. For moxa content analysis, leaves exhibiting uniform weight and leaf area criteria (five replicates for each category) were selected. Samples were oven-dried at 60°C for two days, then pulverized using a high-speed grinder (3 min). The ground material was filtered using a pharmacopoeia sieve with a pore size of 0.85 mm, and the mass of extracted moxa was subsequently weighed. The averages of plant height, leaf area, moxa length, moxa content per unit weight, and moxa content per unit leaf area were subjected to statistical analysis using Excel. This included the calculation of mean values and standard deviations, as well as T-tests and significance analyses.

## 3 Results

### 3.1 Developing repetitive oligo probes from whole-genome sequencing data of *A. argyi*

Initial analyzed QCXA whole-genome sequences using TRF software to identify and extract TRs. After eliminating overlapping TRs, we selected TR arrays that exhibited a copy number ≥50 and a repeat unit length ≥10. This process yielded a total of 72 TR arrays with copy numbers ranging from 50 to 78,181 and repeat unit lengths varying from 1 to 1138 bp. Further analysis conducted using the CD-HIT tool removed redundant TRs (with ≥75% homology), resulting in 64 non-redundant TR sequences. Utilizing Oligo 7 software, we designed 65 oligo sequences based on the core units of these TRs; each sequence ranged in length from 30 to 50 bp (**Supplementary Table S2**).

**Figure 1:**
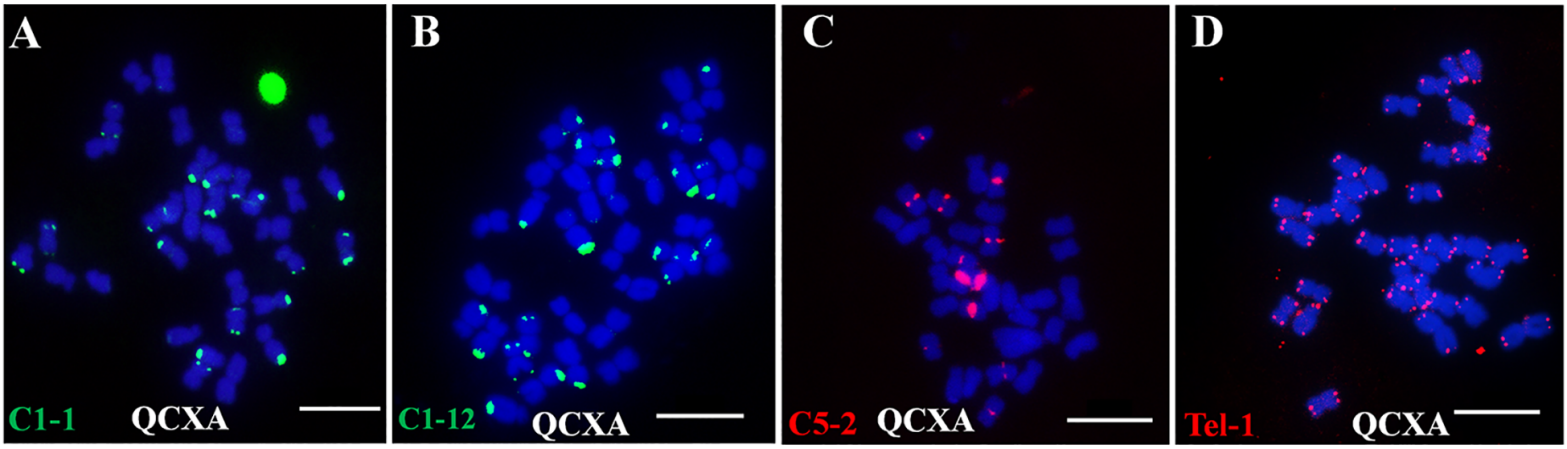
Signals of each probe on the chromosomes of the cultivar Qichun Xiang Ai (QCXA) based on fluorescence *in situ* hybridization (FISH). **(A)** C1-1 (green); **(B)** C1-12 (green); **(C)** C5-2 (red); **(D)** Tel-1 (green). Scale bar: 10 μm.

Following electronic localization screening, 20 highest counts oligo probes were selected for FISH validation. All probes produced clear and stable FISH signals on QCXA chromosomes (**Figure 1**; **Supplementary Figure S1**; **Supplementary Table S3**). Signal distribution patterns revealed remarkable probe specificity: Tel-1 exhibited the most extensive signal coverage and generated signals across all 34 chromosomes. The second most widespread probe, C5-14, displayed signals on 25 chromosomes, while the least widespread probe, C10-7, produced signals on only 4 chromosomes.

Based on their distribution patterns, these probes were classified into five distinct groups: centromere region signal probes (C5-12, C6-21, C1-4, C5-2, C6-2), telomere region signal probes (Tel-1, C1-1), centromere and telomere region signal probes (Co-481, Co-516, C3-1, C1-12), centromere and chromosome arm middle region signal probes (C10-9, C10-10), and telomere and chromosome arm middle region signal probes (C5-14, C10-7, C10-19).

### 3.2 Development of a genome map-based karyotype of *A. argyi*

**Figure 2:**
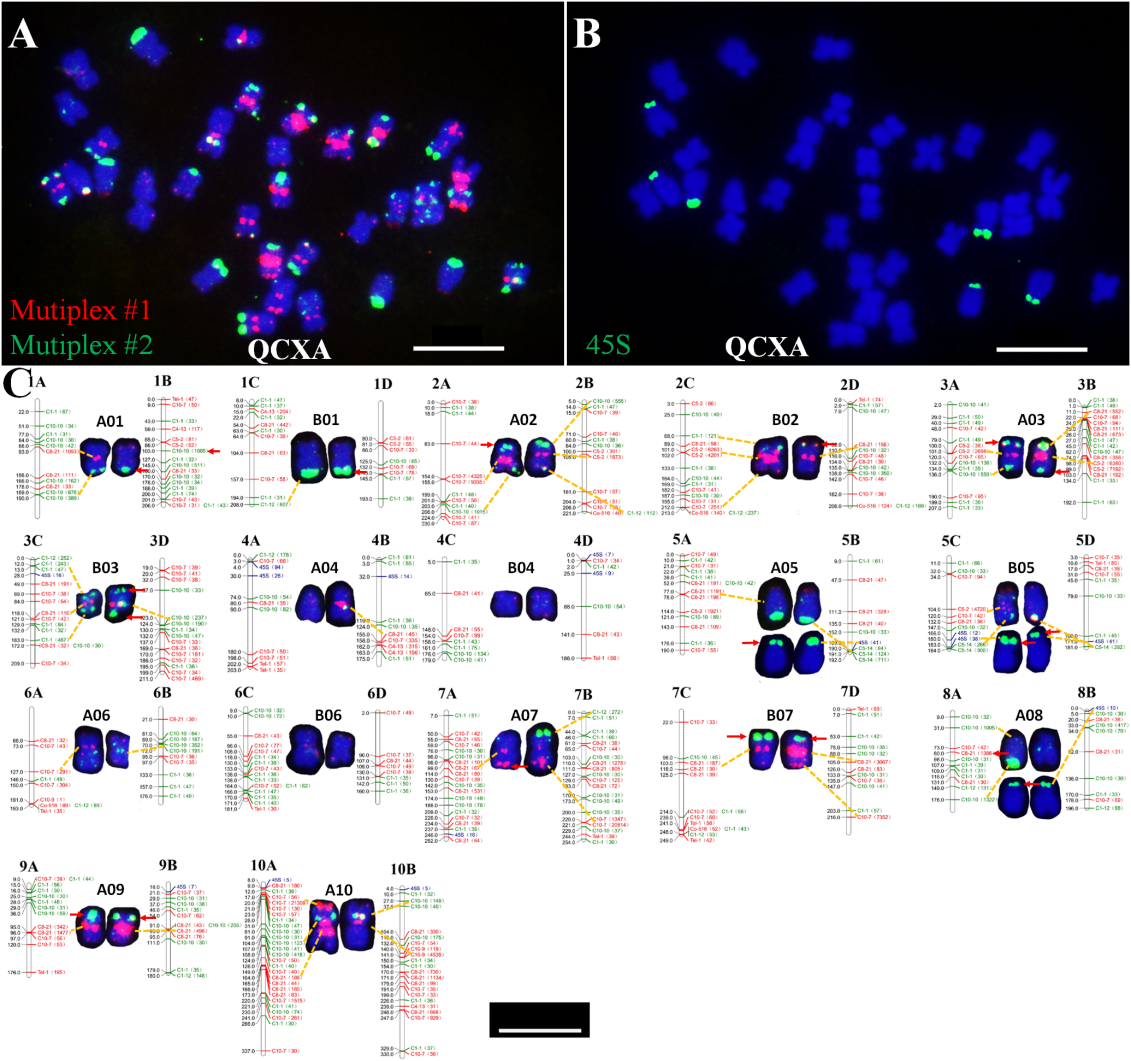
Probe staining and 45S rDNA FISH and karyotype and chromosome plots of the cultivar QCXA. **(A)** Probe staining using Multiplex #1 (red) and Multiplex #2 (green); **(B)** FISH using 45S rDNA (green) as the probe; **(C)** karyotypes corresponding to chromosomes in the sequencing map of QCXA. The yellow dotted line shows the corresponding oligo signals, and the red arrows indicate the non-corresponding oligo signals. Scale bar: 10 μm.

Eleven oligo probes (C8–21, C4–13, C10–9, Co–516, C10–7, C1–1, C5–2, C1–12, C10–10, C5–14, and Tel-1) were selected based on their distinct banding patterns for construction. Through systematic probe combination analysis, two optimized oligo probe cocktails: Multiplex #1 and Multiplex #2 were developed (**Supplementary Table S4**). These cocktails successfully stained QCXA chromosomes (**Figure 2A**), with subsequent 45S rDNA probe FISH (**Figure 2B**) enabling near-complete chromosome identification.

To align the karyotype with the reference genome, electronic localization of the 11 oligo probes and 45S rDNA sequences gene was performed. Each oligo probe was assigned a unique color, code for visualization in the electronic localization map. Then a comprehensive comparison was conducted between FISH-derived signal intensities and chromosomal positions. By incorporating chromosome size data and the maximum similarity of probe distribution between the FISH karyotype and chromosome plots (**Supplementary Table S5**), we constructed a genome map-based karyotype of *A. argyi* that corresponded the chromosomes of QCXA in the karyotype with those in the reference genome (**Figure 2C**).

The finalized karyotype organized chromosomes into homologous pairs A01-A10 and B01-B07. While most probes showed consistent signal patterns between actual karyotypes and reference genome predictions. However, 18 significant mismatched loci were identified (**Figure 2C**). These mismatched loci can be categorized into two distinct situations. The first scenario involves the presence of signals on the chromosome, yet a corresponding site is absent in the sequence map; an example of this is the 45S rDNA signal located at the end of the short arm on chromosome A05. The second scenario pertains to high-frequency repetitions identified in the sequence map that lack corresponding signals on chromosomes within karyotype, such as, C8-21 exhibits 1386 repetitions near the centromeric region on chromosome A08, but no signals are detected on the chromosome during karyotypic analyses. We speculate that these mismatched loci may stem from gap regions within the *A. argyi* genome, or structural chromosomal variations, or heterozygosity between plant analyzed in this study and sequenced plant.

Key chromosomal features in the karyotype included: Chromosome A10 was the largest, with strong red and green oligo signals at both centromere regions. Bright red signals were observed at the terminus of the short arm of chromosome A10 and near the centromere region of its long arm. Chromosomes A05, B05, and A08 contained 45S rDNA sites at their short arm ends; additionally, the chromosome A05 pair and one chromosome A08 showed oligo signals in the terminal regions of the long arm. Distinct red oligo signals were observed in the centromere regions of chromosomes A02, B02, A03, B03, A07, B07, and A09; however, the intensity and size of these signals varied between chromosomes. Specifically, chromosomes A02, A03, and B03 exhibited green oligo signals at both ends, whereas B02 exhibited green oligo signals solely at its short arm ends; furthermore, A07 and B07 displayed red signals at their long arm ends. The terminal ends of both chromosomal arms of A01 and B01 exhibited distinct green signals; notably, one chromosome A01 exhibited a red signal within its centromere region. Chromosomes A06 and B06 exhibited weak green or red signal patterns around their centromeres and scattered signal distributions on their arms. Only one chromosome A04 had a strong centromere band, whereas both chromosomes A04 and B04 had weak red signals at their ends.

### 3.3 Chromosomal distribution of the oligo probes

Based on the newly established QCXA karyotype, we performed FISH analysis of chromosome slides of the same tissue culture seedling to physically map the 20 oligo probes on the chromosomes (**Supplementary Figures S2–S5**). The analysis revealed several key distribution patterns.

**Figure 3:**
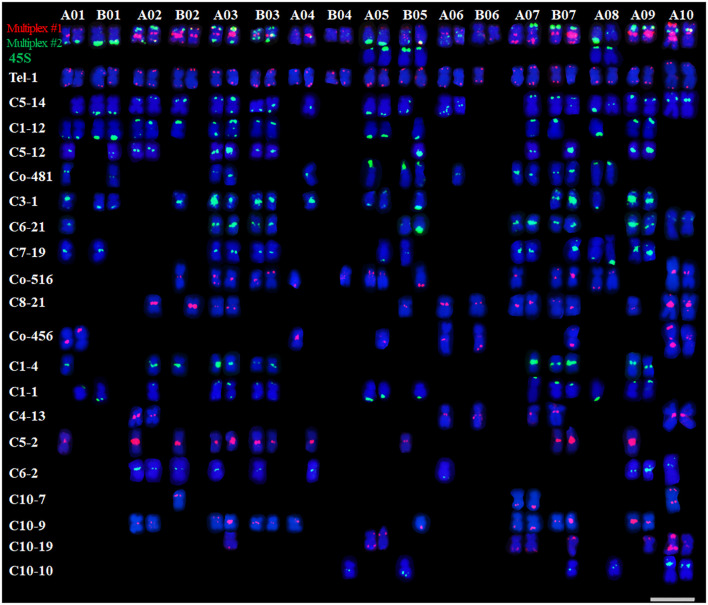
Karyotypes of the distribution of all 20 oligos on QCXA chromosomes by oligo probe staining using Multiplex #1 (red) and Multiplex #2 (green) and sequential FISH using 45S rDNA (green) probes. Scale bar: 10 μm.

Probe Distribution Heterogeneity: High-density chromosomes (≥10 probes) A01, A02, B02, A03, B03, A05, B05, A07, B07, 9A, and 10A. Low-density chromosomes (<10 probes) B01, A04, B04, A06, B06 and A08. Interchromosomal Variation: The repetitive sequence probes displayed markedly uneven chromosomal distributions, with signal density varying up to 6-fold between chromosomes (B04 vs B07). Intrachromosomal Heterozygosity: Most probes (85%) exhibited differential hybridization patterns between homologous pairs. Particularly, probe C3-1 showed monoallelic signals on A01, B02, A04, and B05 homologues (**Figure 3**), indicating substantial heterozygosity in QCXA’s chromosomal architecture.

This comprehensive physical mapping provides the first genome-wide visualization of oligo probe distributions in *A. argyi*, establishing a framework for future comparative genomic studies.

### 3.4 Karyotype analysis of WALs-9, AGQA, and AYBA

**Figure 4:**
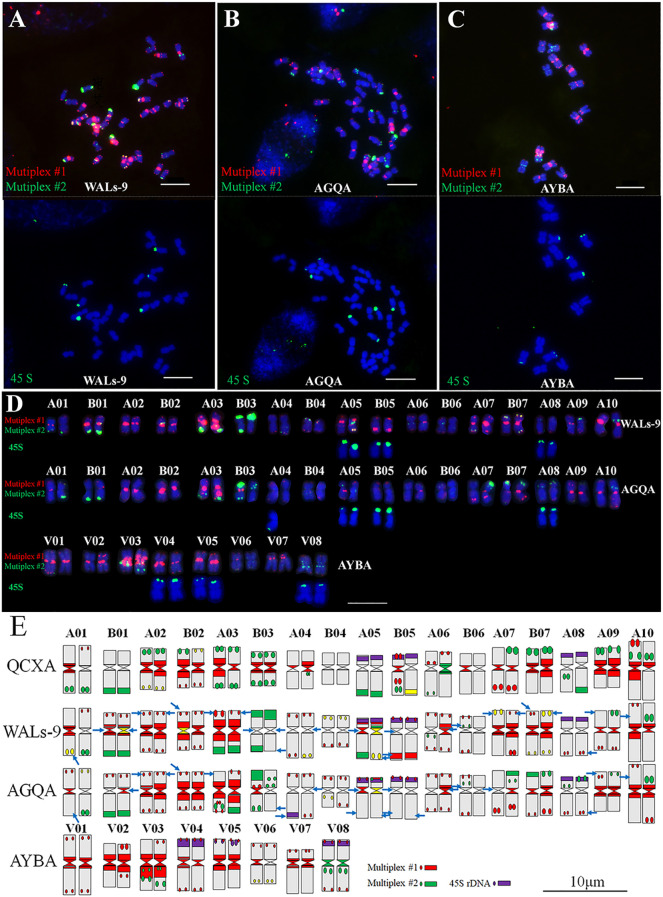
Sequential FISH karyotypes of WALs-9, AGQA and AYBA and their comparison with the karyotype idiogram of QCXA. **(A–C)** Probe staining of WALs-9, AGQA and AYBA using Multiplex #1 (red) and Multiplex #2 (green) and sequential FISH using the 45S rDNA probe (green); **(D)** karyotypes of WALs-9, AGQA and AYBA; **(E)** idiograms of the karyotypes of QCXA, WALs-9, AGQA and AYBA. Blue arrows indicated inconsistent signals compared to QCXA. Bar=10 μm.

Multiplex #1 and Multiplex #2 were used in conjunction with 45S rDNA FISH to analyze the chromosomes of WALs-9 (**Figure 4A**), AGQA (**Figure 4B**), and AYBA (**Figure 4C**) and construct their karyotypes (**Figure 4D**, **Supplementary Table S5**). Both WALs-9 and AGQA possessed 34 chromosomes, including an exceptionally large chromosome 10A; AYBA had 16 chromosomes. 61 major probe signal sites were cataloged within the QCXA karyotype and identified 25 sites (41.0%) in WALs-9 that did not correspond with those in QCXA. In AGQA, 21 sites (34.4%) did not match the QCXA karyotype. These observations suggest that a significant proportion of repetitive sequence distribution sites in WALs-9 and AGQA are either similar or identical to those present in QCXA (**Figure 4E**). The AYBA chromosomes exhibited a diverse array of banding patterns, enabling effective identification of the cultivar’s chromosomes. The 45S rDNA sites in AYBA were predominantly located on the short arms of chromosomes V04, V05, and V08. This distribution partially aligns with the homologous groups identified in AGQA. The analysis of oligo distribution revealed strong or relatively strong red centromere signals on chromosomes V02, V03, and V07; conversely, chromosome V06 displayed weak probe signals (**Figure 4**).

### 3.5 Chromosome pairing behavior of *A. argyi*

**Figure 5:**
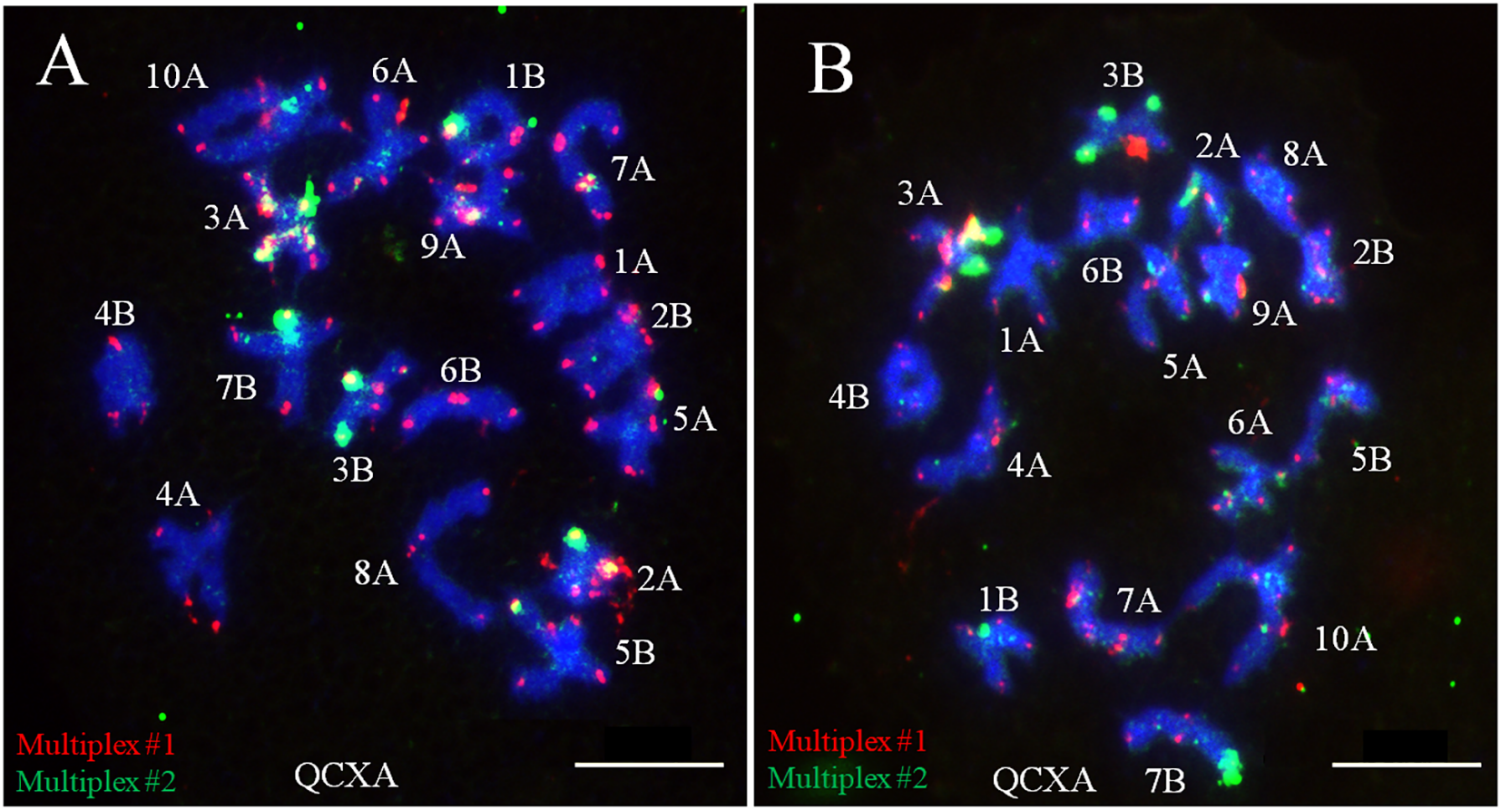
Meiotic chromosome pairing analysis from two different cells **(A, B)** in WALs-9 and their probe staining using Multiplex #1 (red) and Multiplex #2 (green).

To elucidate the chromosome pairing behavior of the allopolyploid *A. argyi*, the chromosomal meiotic behavior of WALs-9 was examined through probe staining. A statistical assessment of monovalent, bivalent, and multivalent chromosomes across 50 cells was conducted. At the diakinesis of meiotic division within PMCs, all 34 chromosomes of WALs-9 paired bivalently with an average configuration of 17II; no trivalent or tetravalent chromosome pairing was detected (**Supplementary Table S6**). Furthermore, chromosomes exhibiting similar or identical banding patterns tended to pair together (**Figure 5**), indicating that WALs-9 may be a highly diploidized special allotetraploid.

### 3.6 Creation and identification of octoploid *A. argyi* with long moxa

**Figure 6:**
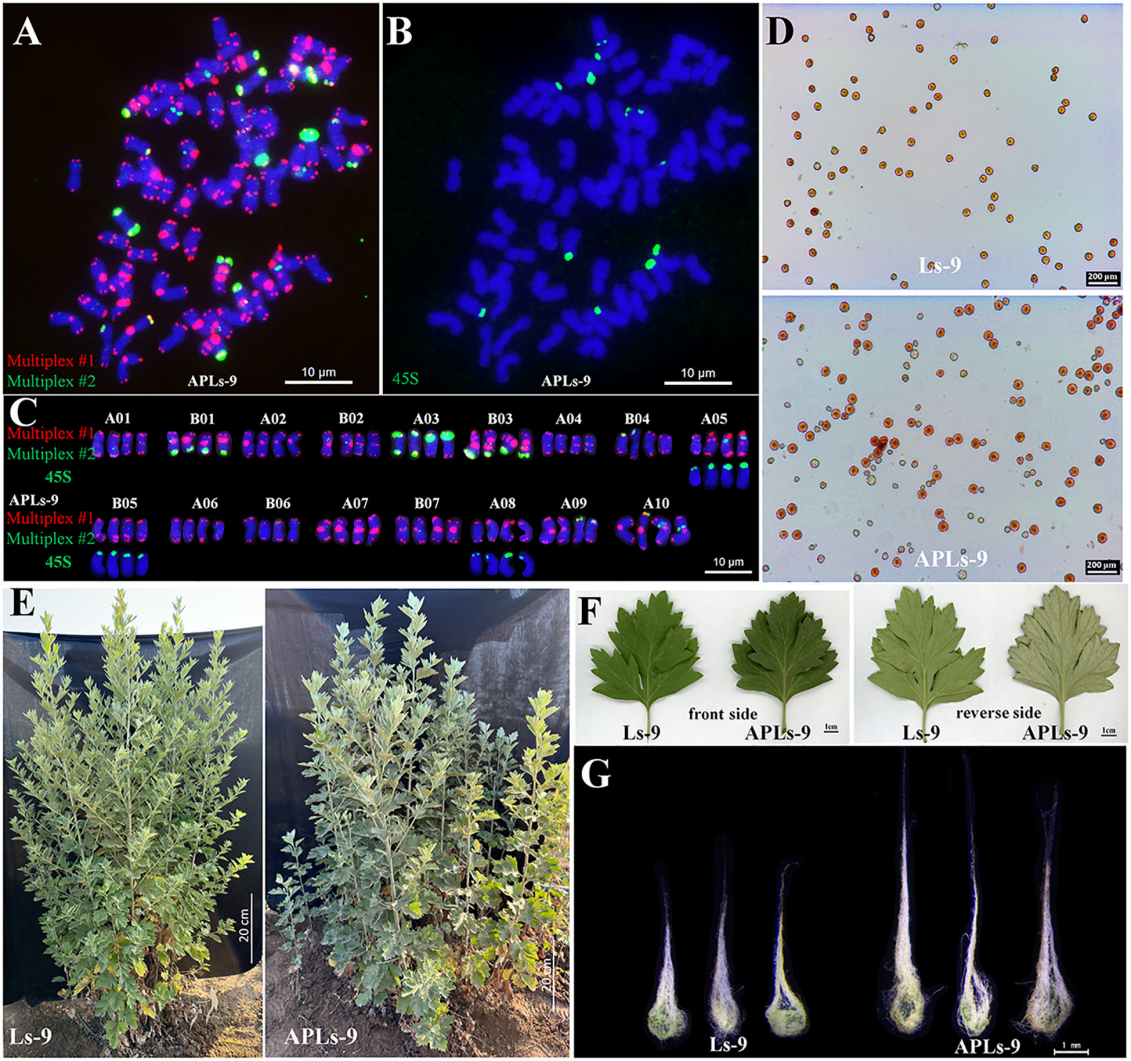
Karyotype identification of APLs-9 **(A–C)** and comparison of APLs-9 with Ls-9 in terms of pollen fertility **(D)**, plant type **(E)**, leaves **(F)**, and moxa length **(G)**. The probe and signal colors in **(A–C)** are the same as those in **Figure 2**.

WALs-9 was treated with colchicine to induce chromosome doubling, resulting in the formation of octoploid *A. argyi* (APLs-9). Analyses utilizing Multiplex #1 and Multiplex #2 alongside 45S rDNA FISH (**Figures 6A, B**) demonstrated that APLs-9 had 68 chromosomes. The homologous chromosome pairs 1A-10A and 1B-7B each evolved into four distinct chromosomes, while two pairs exhibited identical banding patterns. The chromosomal banding profiles were consistent with those observed in Ls-9, thereby confirming that APLs-9 represents an octoploid resulting from chromosome doubling (**Figure 6C**). To assess the chromosomal stability among the asexual reproduction lines of octoploid *A. argyi*, the karyotypes of lines were analyzed using Multiplex #1 and Multiplex #2 alongside 45S rDNA FISH. There were no obvious chromosomal number or structural variations within octoploid karyotype during asexual reproduction (**Supplementary Figures S6, S7**), indicating that the inheritance among these lines is relatively stable.

Pollen fertility analysis indicated that APLs-9 had a fertility rate of 55.23%, significantly lower than the 95.70% fertility rate of Ls-9 (**Figure 6D**, **Table 1**). Further phenotypic investigations of Ls-9 and APLs-9 encompassing measurements of plant height, moxa content per unit weight, and moxa length per unit area revealed notable differences between APLs-9 and Ls-9. The average plant height of APLs-9 (111.60 ± 8.39 cm) was significantly shorter than that of Ls-9 (135.38 ± 13.75 cm) (**Figure 6E**).

**Table 1:** Comparison of phenotypic traits and pollen fertility between Ls-9 and APLs-9.

Traits	Ls-9	APLs-9	t-test
Plant height (cm)	135.38 ± 13.75	111.60 ± 8.39	7.35**
Leaf area (cm^2^)	39.67 ± 4.90	35.81 ± 9.05	1.63
Moxa content/weight (g/g)	0.68 ± 0.10	0.66 ± 0.11	0.73
Moxa content/leaf area (mg/cm^2^)	1.03 ± 0.02	1.25 ± 0.03	11.65**
Moxa length (mm)	2.40 ± 0.43	4.23 ± 0.57	-13.92**
Pollen fertility (%)	95.68 ± 1.36	55.23 ± 8.27	15.26**

**indicates a very significant level of difference, p < 0.01.

The upper surface of APLs-9 leaves displayed a darker green hue and greater thickness than those of Ls-9; conversely, the underside of the leaves appeared paler and contained denser moxa (**Figure 6F**). The average moxa content per unit weight was similar between APLs-9 (0.66 ± 0.11 g/g) and Ls-9 (0.68 ± 0.10 g/g) (**Table 1**). However, the average moxa content per unit leaf area was significantly higher for APLs-9 (1.25 ± 0.03 mg/cm²) than for Ls-9 (1.03 ± 0.02 mg/cm²) (**Table 1**). Additionally, APLs-9 had longer moxa, the average moxa length of APLs-9 (4.23 ± 0.57 mm) was about 1.8 times longer than that of Ls-9 (2.40 ± 0.43 mm) (**Figure 6G**).

## 4 Discussion

### 4.1 Development and application of repetitive sequence oligo probes in *A. argyi*

As a medicinally significant species, *A. argyi* has lacked comprehensive karyotype characterization, hindering understanding of its genomic evolution. While He et al. (2024) pioneered FISH karyotyping using seven *Artemisia*-derived oligo probes, their approach suffered from limited resolution and required multiple FISH iterations (He et al., 2024). The chromosome-level genome assembly of *A. argyi* revealed a genome size of 8.03 Gb with 73.59% repetitive sequences (Miao et al., 2022). Based on this reference genome, we designed and developed 20 probes producing robust signals on chromosomes of *A. argyi* in this study. These probes were further combined into high-resolution probe cocktails for efficient, cost-effective chromosomal probe staining under non-denaturing conditions. The probe cocktails were used to construct high-resolution karyotypes of WALs-9, AGQA, and AYBA, leading to the creation and identification of a novel octoploid germplasm. The repetitive sequence oligo probes and staining technology developed in this study provide a technical foundation for the effective identification of chromosomal structural variations and the exploration of chromosome evolution in *A. argyi*.

In the karyotype of *A. argyi*, we observed mismatches between certain repeated sequence sites identified by FISH and their corresponding locations on the sequence map. Similar mismatches have been reported in studies using repeated sequence oligos for crops such as peanut and wheat (Du et al., 2017; Fu et al., 2021; Du et al., 2025). These mismatches may arise from gap regions within the *A. argyi* genome or from heterozygosity between the sequenced *A. argyi* and the plants examined in this study. Structural variations within the chromosomes of *A. argyi* may also contribute to these inconsistencies. Furthermore, it is important to note that the 20 repetitive sequence oligos that were shown to generate signals on *A. argyi* represent only a small fraction of the total oligos designed in this study. In our constructed karyotypes of *A. argyi*, several chromosomes exhibited similar banding patterns, such as A07 and A09. In future work, we will continue screening additional repetitive sequence oligo probes to further enrich chromosome bands based on the methodologies established in this study. Simultaneously, we plan to develop libraries of single-copy sequence oligo probes with the aim of enhancing chromosome identification through chromosome painting techniques.

### 4.2 Genome map-based karyotype application

Genome sequencing provided a reference genome for *A. argyi*. In this study, oligo probes were used for electronic mapping of reference genome sequences and FISH mapping of actual chromosomes to establish a genome map-based karyotype. Most of the repeated sequence loci were consistent between the karyotype and sequence map; however, there were also inconsistent loci, suggesting the existence of gaps in these regions of the genome. Based on banding differences observed in some homologous chromosomes, as well as high pollen fertility and meiosis chromosome pairing behavior, we speculate that *A. argyi* may be a special allotetraploid with a chromosome base of 17. Furthermore, the number of basic chromosomes of *A. argyi* is nominally x = 9 (Pellicer et al., 2010). In the genome of *A. argyi*, ancestral chromosomes 8 and 9 have fused to form chromosome 10 (Miao et al., 2022). Additionally, several species within *Artemisia* exhibit a basic chromosome number of x=8 (He et al., 2024), indicated that this fusion event may have occurred prior to hybridization polyploidization. However, further evidence supporting chromosome fusion in species with a chromosomal base of 8 is still required. Confirmation of the ploidy level in *A. argyi* will require additional methodologies such as hybridization experiments involving wild related species of *A. argyi*, genomic homology analysis, gene expression analysis, and physical localization of repetitive sequences.

The subgenus *Artemisia* encompasses several species, such as *A. argyi*, *A. annua*, *A. tournefortiana*, *A. vulgaris*, *A. chamaemelifolia*, *A. molinieri*, *A. lucentica*, and *A. judaica*. Their chromosome numbers vary considerably and include 2n =16, 18, 34 and 36 (Pellicer et al., 2010). Chromosomal fusion/fission and polyploidy are the main sources of chromosome number evolution (Mitros et al., 2020; Xuan et al., 2022). In this study, we found that *A. argyi* cultivars QCXA, WALs-9, and AGQA all had 34 chromosomes; moreover, their chromosomal banding patterns displayed significant similarities, suggesting a common ancestral lineage. The chromosome base number of *A. vulgaris* AYBA was x = 8. Notably, most of the probes derived from QCXA produced signals in AYBA, and 45S rDNA sites were distributed on homoeologous groups 5 and 8, indicating a potential evolutionary relationship between the genomes of *A. vulgaris* and *A. argyi*. However, the reported nominal base chromosome number of *A. argyi* is x =9 or x =17 (Pellicer et al., 2010). Species with a chromosome base number of 8 might be derived from descendent dysploidy caused by chromosomal fusion (Vallès and Siljak-Yakovlev, 1997). Furthermore, repeat sequences are not uniformly distributed within genomes and tend to vary significantly; thus, accurately identifying homoeologous groups remains challenging. Developing additional oligo probes based on single-copy sequences is crucial for establishing a consistently defined karyotype, which will facilitate further studies of the evolutionary relationships among species within the subgenus *Artemisia* (Han et al., 2015; Yu et al., 2021).

### 4.3 Creation of polyploid *A. argyi* and its application potential in breeding

The production of moxa and *A. argyi* extracts is directly dependent on the plant’s biological yield. Polyploidy not only induces alterations in the plant genome but also impacts gene expression, including changes in genome size and structure, upregulation or downregulation of gene expression, and modifications in DNA methylation patterns (Lavania et al., 2012; Parisod et al., 2010; Yu et al., 2010). Artificially induced polyploidy is frequently associated with leaf thickening, deepening of leaf color, and enlargement of plant organs. Moreover, the contents of certain nutrients or secondary metabolites tend to be significantly greater in polyploid plants due to the elevated gene dosage (Thong-On et al., 2014; Vyas et al., 2007). In this study, a novel octoploid *A. argyi* germplasm with significantly greater moxa length and moxa content per unit leaf area, i.e., APLs-9, was developed. This advancement provides material for enhancing the quality and increasing the yield of moxa. Polyploidization has been observed in various medicinal plants and can alter phenotypes and medicinal components. In ginseng, polyploid adventitious roots enhance the production of secondary metabolites (Kim et al., 2004). In tetraploid and diploid plants of *Astragalus membranaceus*, N^+^ implantation induces changes in multiple biochemical indexes; self-protection and stress resistance are higher in tetraploid plants than in diploid plants (Zhang et al., 2011). Polyploidy results in modifications of metabolites and resistance due to variations in gene dosage effects, genomic structure, epigenetic modifications, and enzyme activities (Song and Chen, 2015). The moxa and leaf oil of *A. argyi* have high medicinal value. Additional studies are needed to determine if the medicinal components of octoploid *A. argyi* are altered.

Chromosome doubling also produced adverse traits, such as reduced plant height and lower fertility. Furthermore, disruption of autopolyploid chromosome synapsis may result in decreased pollen fertility. Artificially created polyploid materials are also susceptible to chromosome loss and diploidization during sexual reproduction (Zhang et al., 2013). Therefore, during the process of sexual reproduction (seed reproduction), there is a possibility that the chromosome number of offspring may vary, potentially reducing beneficial traits. Specialized approaches are needed to address the challenges posed by polyploidy. Fortunately, *A. argyi* can reproduce asexually. Chromosome stability analysis of octoploid *A. argyi* during asexual reproduction revealed no significant chromosomal number or structural variations within its karyotype; moreover, the inheritance patterns among lines produced through asexual reproduction remained relatively stable. Thus, asexual propagation can preserve this material and its advantageous traits. To improve plant height while preserving the long-moxa characteristics of octoploid *A. argyi*, an appropriate next step would be to establish an aneuploidy system via hybridization or self-crossing aimed at improving both the plant height and biological yield of *A. argyi* for practical applications through asexual propagation. This polyploid breeding platform, combined with high-resolution karyotyping system, provides unprecedented opportunities for *A. argyi* cultivar improvement. The APLs-9 germplasm represents a significant advance in medicinal plant breeding, offering both scientific insights and practical applications for moxa production.

## 5 Conclusions

This study designed 20 novel repetitive sequence oligo in *A. argyi* based on a reference sequence and developed two optimized probe cocktails probe cocktails—Multiplex #1 and Multiplex #2—that facilitate highly efficient chromosome staining under non-denaturing conditions. By combining probe staining with 45S rDNA FISH, we established the first genome map-based karyotype of *A. argyi*; characterized chromosomal features across four cultivars QCXA, WALs-9, AGQA, and AYBA; Identified the chromosomal distribution patterns of the newly developed probes through FISH localization studies; and obtained evidence indicating that *A. argyi* may be a special allotetraploid species with a chromosome base number of 17. Furthermore, a novel octoploid *A. argyi* germplasm with significantly enhanced moxa length and moxa content per leaf area was created and identified. These findings provide both technical tools (probes/karyotyping system) and genetic resources (polyploid germplasm) for advancing *A. argyi* breeding programs focused on moxa quality improvement.

## Data Availability

The original contributions presented in the study are included in the article/**Supplementary Material**. Further inquiries can be directed to the corresponding author/s.
