# Supplementary material for: Establishment of a genome map-based karyotype of Artemisia argyi and identification of a new octoploid
Source: Front Plant Sci. 2025 Jun 25;16:1621415. doi: 10.3389/fpls.2025.1621415 (PMC12238084; doi:10.3389/fpls.2025.1621415)
Supplement: Supplementary file 1 [file DataSheet1.pdf]

TABLE S1 *Artemisia* cultivars used in this study

Number	variety name	Provenance	Section/type	Provider
QCXA	Xiang Ai	Qichun, Hubei, China	*A. argyi*	Hubei University of Chinese Medicine
WALs-9	Wan Ai	Nanyang, Henan, China	*A. argyi*	Institute of Chinese Herbal Medicines, Henan Academy of Agricultural Sciences
AGQA	Qi Ai	Anguo, Hebei, China	*A. argyi*	Nanjing Agricultural University
AYBA	Bei Ai	Anyang, Henan, China	*A. vulgaris*	Institute of Chinese Herbal Medicines, Henan Academy of Agricultural Sciences

TABLE S2 Repetitive sequences oligos from the *A. argyi* genome

	Oligo	Sequence of oligos	Length	Total-copy-number
1	C10-7	CGTGTGATCCCACGTCATCTCGACTCGTTCGTTTAACATCT	41	78181
2	C1-4	CGATGACCTACGACCAAGGTTCCGTGACCTTG	32	66141
3	Chr10_2-10	GCATTAGGGACAACGGCTATCATCATGTAAAGGGCAAT	38	22326
4	Chr10_2-10	AGACAACGGATCTCATCATGTAAGTGACAATTGCTCATAT	40	22326
5	C8-21	TTATGACCCGAATAAAAATATAACCGATTTAATGAAACC	39	10599
6	C10-19	ACGGATGAGTCGATATGTTATGGTTGCACATTAGACGTTCA	41	9848
7	Chr10_1-10	GGCCACTTTATGCTTAATTGTATGTGACTAAATACCCAAA	40	8783
8	C6-21	CTTGGAGGCGAAATAATAAAATCTGGAGTTAGTTT	35	8414
9	C1-12	TTGATAACGGCTATTTCTATGACAGTTTTATTTGCGCTTC	40	6662
10	C5-2	GTCACGGAACCATGGTCGTAGGTCATCCA	29	6428
11	Chr10_1-1	TGGCCAAGACCCAGCTAGAACCAGGTAAGACTCGGCTGTTC	41	6298
12	Co-456	GAACAATGTTAGGAAGAAAAATGGATTGCCGCACGTCAAG	40	5193
13	Contig_23456-1	TTTTTCCTTCTTAGTCGCCTTTGGTGGTTCCGAGGTC	37	5193
14	C3-1	ATTATAACCGGTTTAATTAATGAGACGAACTAATCCA	37	4864
15	C5-12	ACGTAAAAAAAACAGACGAAAACGTAAAAAAAACAGACGAAA	42	4241
16	C6-2	CGTTTTTGTCCGTTTTTCTTACGTTTTTGTCCGTTTTTCTTA	42	2907
17	C7-19	AAGTAATTCTAGCCACACATTTCAAATTAGGAGGGGA	37	2894
18	Chr10_1-8	ATATATATATATATATATATATATATATATATATATATATAT	42	2671
19	C10-9	GACACAATTGACAACAAAAATGCCAAACTAATAGGTAAAA	40	2531
20	C5-14	CATCGACCATTTTTAATTTCAACTTGTCCTCAAACCAATA	40	1983
21	C1-1	TTGTTATGGATAAGCATAACATGTTAAACGGATGAGTCGAG	41	1758

22	Contig_22516-1	ATGACAGTTTTTATTTGCGCTTGATAACATTTAGTATATT	40	1628
23	Co-516	TTTTACTAGGGCGTCGGATTGAGGTGATATTTTTGACATT	40	1628
24	Chr10_1-12	TAAAAACGTGTGTAAAAACGTGTGTAAAAACGTGTGTAAAAACGTGTG	48	1500
25	Chr5_3-2	TTCGATACCTAATTTCTTCACATGTGAAGGTA	32	836
26	Co-481	GACGATTCCCAGGCCGACGTTGAACGACGAGGCCTATTACGAAGC	45	798
27	C4-13	ATTTTATATTACTTACAAGCTCGAGAACTCAACAATAACT	40	693
28	Chr5_4-1	AGAATTAATTTTTTGTTAAGGCTCTTGGGCTCTAACTGGATTTCC	45	680
29	Chr9_1-7	CAACCAATGCTATTATAGATGTACTTATTAAAATTTGCCA	40	542
30	Chr4_3-2	CTATAATGGGTATAACTTGGGCTACAGGGCTCCGTTTCA	39	444
31	Chr7_4-37	CGTTCTCCTCCACCGCACCGTCGATCATAAACCGTTACCA	40	410
32	Chr3_2-2	ATTATTTAAGTATACTAAAAGTTTCTTCACGAGTGAAGAATATCG	45	367
33	Chr1_2-4	ACCACCACCACCACCACCACCACCACCACCACCACCACCACCACCACCACCACC	54	365
34	Chr3_2-13	TGCGCCTTGAGTTCGTCCATGTTAGTTAAAGTTTCAGATT	40	356
35	Chr3_2-13	ATTTTAGACACATATAGGATTAGAGGACCACCGGGATCAC	40	356
36	Chr3_2-13	TTTACCAGCAAACACAAGACCGAAAGCGAAGAATCTACC	39	356
37	Chr6_3-5	TTTTACGTTTTCATCTATTTTTTTACGTTTTCATCTATTT	40	283
38	Chr7_1-27	TGGAGGTGTAACACCGGGTGATGGAGCTGGTGTGTCTGGTGTAGG	45	258
39	Chr7_3-14	TGACTAGCAACTAATTCCGCCCACTCCTTCTAAAACCGA	39	238
40	Chr3_3-13	TATAGGGTGCGTTTGATTTATAAGTGGGATTGGAATTG	38	226
41	Chr2_3-8	TTGGCATTTGAGGTAAGTTTTGGGTAATTCCGGCGAG	37	218
42	Chr10_1-23	AACACTTGGCCCATAGTTAGGAGTTGCATCTTTCAAA	37	179
43	Contig_22133-1	GGTTCTTCTACATACAGTTCTAGCGGCATAGTAAAGG	37	174

44	Chr7_3-11	ATTATCGAGAAACTTCAGATTATCGAGAAACTTCAG	36	172
45	Chr10_2-4	TTTTGAATTTTCCACTTGTCATCATCTTTTGTTGCTTTA	39	159
46	Chr7_1-25	TGGCCCAGAACAGTCAACCTAAATTAACCAAGTATATG	38	156
47	Chr7_4-39	TATGTTGTGTAAGTGAGTTGGTGTTTGTTAGAATACGCGA	40	146
48	Chr4_2-17	GGTTTGGCCGGAATTAGCCGGGTTTGGCCTGAACTAAGCCG	41	118
49	Contig_5246-1	ATCTATTGGGCTTATGTTTGTGTTATAGTAATGTAGTTGG	40	110
50	Chr10_1-18	GAATGTGTTTGCCTGATTGTGCTCGTGATGTACTAGAAAA	40	89
51	Chr7_2-4	TAAGAACTTCGTCACATGTGAAAATATATGTCTCGTAGCTTTT	43	88
52	Contig_4292-1	GCGTGAACAAACAGAAGCAGATTACACAGAGGAAGACAAG	40	78
53	Chr9_2-16	GTTTATTTCATGTTTAATTAGGCAGATCTATTTGATTAT	39	73
54	Chr3_2-14	TAAAGAAGAGAAATCTAAGAAACAACAAACACGTTT	36	63
55	Chr5_1-12	TCGATAGGATTGTAACGTGGGCAAACTCGGTAACCAAGAA	40	63
56	Chr5_1-12	GAAATTTTGCACCTAACTCGAAAACAACCTGATGAAC	37	63
57	Chr1_1-3	TAATTAACCTATGATTATCCCCATGAAACCCACCAAG	37	62
58	Chr5_1-3	GTAACATCGTTTCAATCGGGCTATTTAATAACTAACT	37	61
59	Chr4_4-3	TTAGAGGCCTAACTGAGATGCACTGCTATTTGATTTG	37	55
60	Chr6_1-20	TCAAACAAAGAGATGATGCGGTTAAAGAAGTTGGAAT	37	53
61	Chr6_1-20	CAAATATCAGTACTAGAACATCACTTGGCTGCTAAGG	37	53
62	Chr2_2-20	TTCGTCATCATCTCTACTCTAACATACATCAAAGCCCCAA	40	52
63	Chr7_3-1	CCAGTTTACAAGTCTCCACCACCTCCC	27	51
64	Tel	TTTAGGGTTTAGGGTTTAGGGTTTAGGGTTTAGGGTTTAGGGTTTAGGGTTTAGGG	56	51
65	Chr4_4-10	AAGTAAGTTTAATGCATTGAGAAAGCCCTTATTGTCTAG	39	50

TABLE S3 Details of the new oligo probes from the *A. argyi*

Oligos	Sequences of oligos
C6-2	CGTTTTTGTCCGTTTTTCTTACGTTTTTGTCCGTTTTTCTTA
C10-7	CGTGTGATCCCACGTCATCTCGACTCGTTCGTTTAACATCT
C5-12	ACGTAAAAAAAACAGACGAAAACGTAAAAAAAACAGACGAAA
C5-2	GTCACGGAACCATGGTCGTAGGTCATCCA
Co-456	GAACAATGTTAGGAAGAAAAATGGATTGCCGCACGTCAAG
C3-1	ATTATAACCGGTTTAATTAATGAGACGAACTAATCCA
C10-19	ACGGATGAGTCGATATGTTATGGTTGCACATTAGACGTTCA
C1-1	TTGTTATGGATAAGCATAACATGTTAAACGGATGAGTCGAG
C1-12	TTGATAACGGCTATTTCTATGACAGTTTTATTTGCGCTTC
C10-10	GGCCACTTTATGCTTAATTGTATGTGACTAAATACCCAAA
C8-21	TTATGACCCGAATAAAAATATAACCGATTTAATGAAACC
C7-19	AAGTAATTCTAGCCACACATTTCAAATTAGGAGGGGA
C5-14	CATCGACCATTTTTAATTTCAACTTGTCCTCAAACCAATA
Co-481	GACGATTCCCAGGCCGACGTTGAACGACGAGGCCTATTACGAAGC
C4-13	ATTTTATATTACTTACAAGCTCGAGAACTCAACAATAACT
C10-9	GACACAATTGACAACAAAAATGCCAAACTAATAGGTAAAA
C1-4	CGATGACCTACGACCAAGGTTCCGTGACCTTG
Co-516	TTTTACTAGGGCGTCGGATTGAGGTGATATTTTTGACATT
C6-21	CTTGGAGGCGAAATAATAAAATCTGGAGTTAGTTT
Tel-1	TTTAGGGTTTAGGGTTTAGGGTTTAGGGTTTAGGGTTTAGGGTTTAGGGTTTAGGG

TABLE S4 Oligo probes cocktails of *A. argyi* developed in this study

Probe cocktails	Name of oligo	Concentration (ng/μL)	Volume (μL)
Multiplex #1	TAMRA-C8-21	0.125	5
	TAMRA-C4-13	0.125	5
	TAMRA-C10-9	0.125	5
	TAMRA-Co-516	0.125	5
	TAMRA-C10-7	0.125	5
	TAMRA-C5-2	0.125	5
	TAMRA-Tel-1	0.125	5
Multiplex #2	FAM-C1-1	0.125	5
	FAM-C1-12	0.125	5
	FAM-C10-10	0.125	5
	FAM-C5-14	0.125	5

TABLE S5 Chromosome information of QCXA, WALs-9, AGQA and AYBA

QCXA			WALs-9			AGQA			AYBA		
Chr.	Chr. Average length	Arm ratio	Chr.	Chr. Average length	Arm ratio	Chr.	Chr. Average length	Arm ratio	Chr.	Chr. Average length	Arm ratio
A01	5.89±0.54	1.25±0.16	A01	5.55±0.51	1.20±0.24	A01	5.62±0.46	1.21±0.13	V01	6.92±0.33	1.15±0.06
B01	5.26±0.40	1.56±0.27	B01	5.54±0.43	1.34±0.27	B01	5.43±0.19	1.22±0.20	V02	5.73±0.12	1.27±0.07
A02	5.17±0.48	1.37±0.18	A02	5.00±0.41	1.17±0.11	A02	5.71±0.36	1.41±0.14	V03	6.84±0.26	1.22±0.03
B02	5.07±0.15	1.28±0.17	B02	5.06±0.42	1.09±0.12	B02	5.26±0.47	1.19±0.20	V04	6.09±0.29	1.09±0.05
A03	5.12±0.27	1.41±0.13	A03	5.59±0.47	1.17±0.12	A03	5.78±0.23	1.05±0.12	V05	5.73±0.34	1.24±0.17
B03	4.75±0.41	1.25±0.19	B03	5.05±0.52	1.33±0.20	B03	5.79±0.17	1.30±0.31	V06	5.31±0.28	1.21±0.26
4A04	4.80±0.42	1.38±0.09	4A04	5.45±0.33	1.14±0.11	4A04	5.33±0.30	1.38±0.13	V07	5.65±0.61	1.95±0.18
4B04	4.52±0.28	1.49±0.39	4B04	3.90±0.48	1.15±0.18	4B04	4.87±0.24	1.29±0.03	V08	5.80±0.38	1.30±0.12
A05	5.83±0.53	1.78±0.13	A05	5.95±0.43	1.69±0.21	A05	5.65±0.37	1.99±0.27			
B05	5.24±0.40	2.02±0.33	B05	5.72±0.32	1.87±0.38	B05	5.20±0.21	2.03±0.15			
A06	5.45±0.14	1.35±0.35	A06	4.72±0.55	1.30±0.10	A06	5.75±0.42	1.22±0.22			
B06	5.53±0.28	1.15±0.08	B06	5.32±0.41	1.26±0.13	B06	5.40±0.29	1.36±0.35			
A07	5.27±0.22	1.22±0.20	A07	6.00±0.42	1.52±0.14	A07	6.18±0.31	1.30±0.20			
B07	5.45±0.31	1.25±0.20	B07	6.03±0.49	1.23±0.20	B07	6.05±0.45	1.32±0.37			
A08	4.91±0.08	1.73±0.48	A08	4.80±0.58	1.97±0.32	A08	5.41±0.42	2.07±0.31			
A09	5.53±0.27	1.28±0.19	A09	4.98±0.37	1.27±0.29	A09	5.38±0.16	1.34±0.18			
A10	7.97±0.31	1.35±0.21	A10	7.76±0.39	1.29±0.22	A10	7.96±0.33	1.09±0.13			

TABLE S6 Meiotic terminal phase chromosomes of WALs-9

Cells	Monovalent (I)	Bivalent (II)	Trivalent (III)	Tetravalent (IV)
Cell-1	0	17	0	0
Cell-2	0	17	0	0
Cell-3	0	17	0	0
Cell-4	0	17	0	0
Cell-5	0	17	0	0
Cell-6	0	17	0	0
Cell-7	0	17	0	0
Cell-8	0	17	0	0
Cell-9	0	17	0	0
Cell-10	0	17	0	0
Cell-11	0	17	0	0
Cell-12	0	17	0	0
Cell-13	0	17	0	0
Cell-14	0	17	0	0
Cell-15	0	17	0	0
Cell-16	0	17	0	0
Cell-17	0	17	0	0
Cell-18	0	17	0	0
Cell-19	0	17	0	0
Cell-20	0	17	0	0
Cell-21	0	17	0	0
Cell-22	0	17	0	0
Cell-23	0	17	0	0
Cell-24	0	17	0	0
Cell-25	0	17	0	0
Cell-26	0	17	0	0
Cell-27	0	17	0	0
Cell-28	0	17	0	0
Cell-29	0	17	0	0
Cell-30	0	17	0	0
Cell-31	0	17	0	0
Cell-32	0	17	0	0
Cell-33	0	17	0	0
Cell-34	0	17	0	0
Cell-35	0	17	0	0
Cell-36	0	17	0	0
Cell-37	0	17	0	0
Cell-38	0	17	0	0
Cell-39	0	17	0	0
Cell-40	0	17	0	0
Cell-41	0	17	0	0
Cell-42	0	17	0	0
Cell-43	0	17	0	0
Cell-44	0	17	0	0
Cell-45	0	17	0	0
Cell-46	0	17	0	0
Cell-47	0	17	0	0
Cell-48	0	17	0	0
Cell-49	0	17	0	0
Cell-50	0	17	0	0

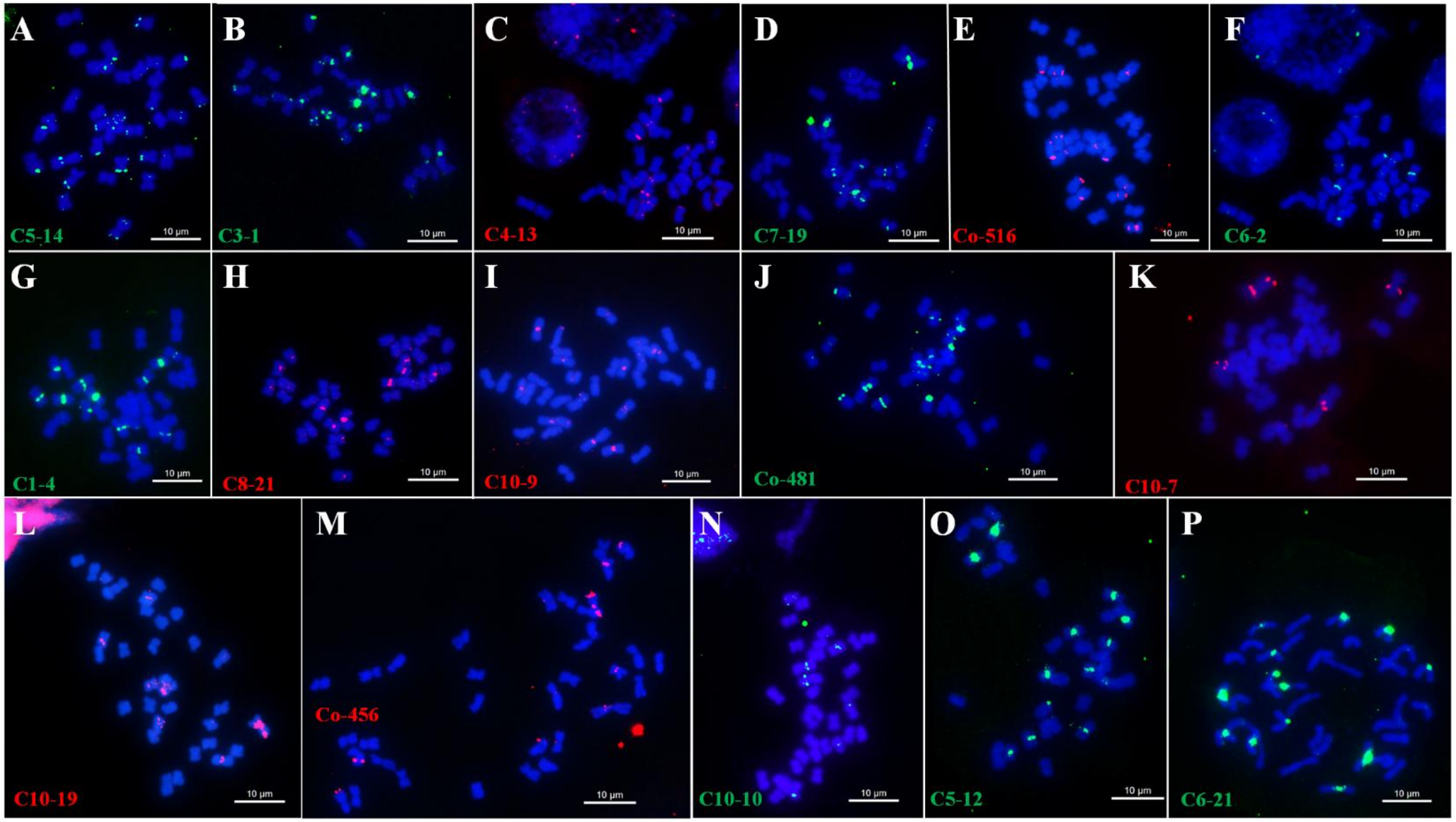

FIGURE S1. Signals of each probe on the chromosomes of the cultivar QCXA based on FISH
(A) C5-14 (green); (B) C3-1 (green); (C) C4-13 (red); (D) C7-19 (green); (E) Co-516 (red); (F) C6-2 (green); (G) C1-4 (green); (H) C8-21 (red); (I) C10-9 (red); (J) Co-481 (green); (K) C10-7 (red); (L) C10-19 (red); (M) Co-456 (red); (N) C10-10 (green); (O) C5-12 (green); (P) C6-21 (green).

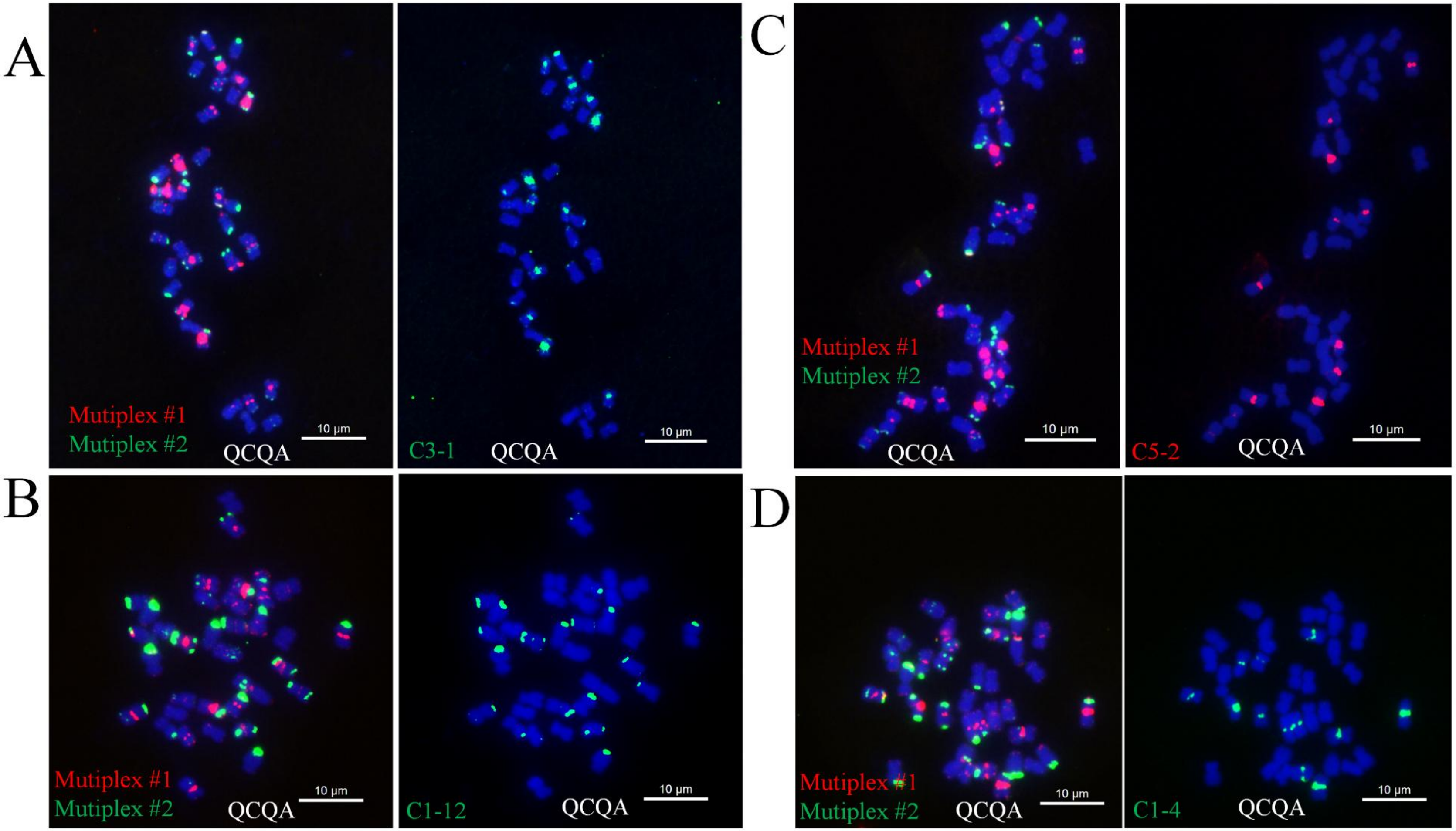

FIGURE S2 Sequential FISH localization of probe C3-1 (A, green), C1-12 (B, green), C5-2 (C, red), and C1-4 (D, green). The left column is probe staining using Multiplex #1(red) and Multiplex #2 (green), The left column is FISH localization of probes.

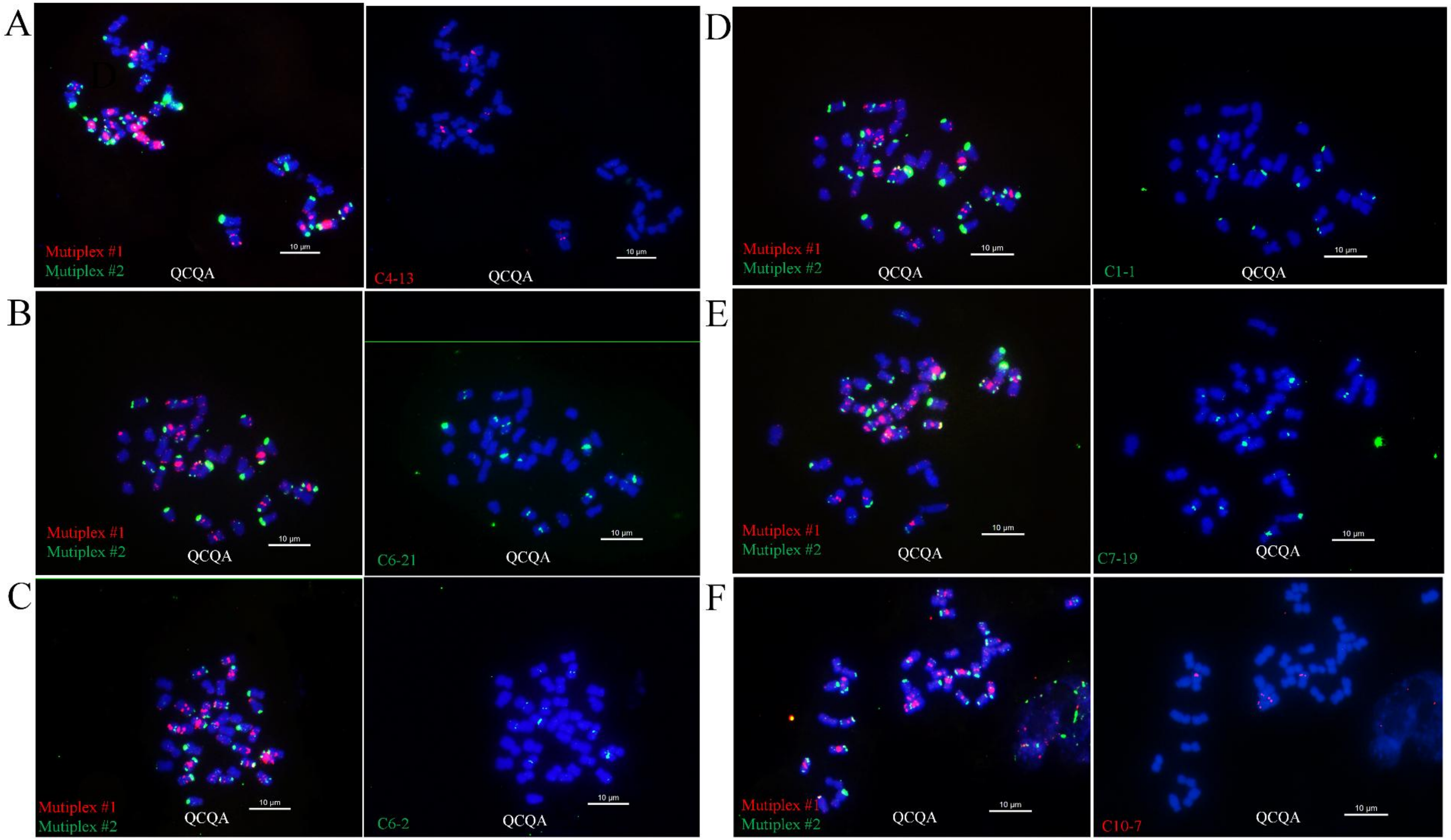

FIGURE S3 Sequential FISH localization of probe C4-13 (A, red), C6-21 (B, green), C6-2 (C, green), C1-1 (D, green), C7-19 (E, green), and C10-7 (F). The left column is probe staining using Multiplex #1(red) and Multiplex #2 (green), The left column is FISH localization of probes.

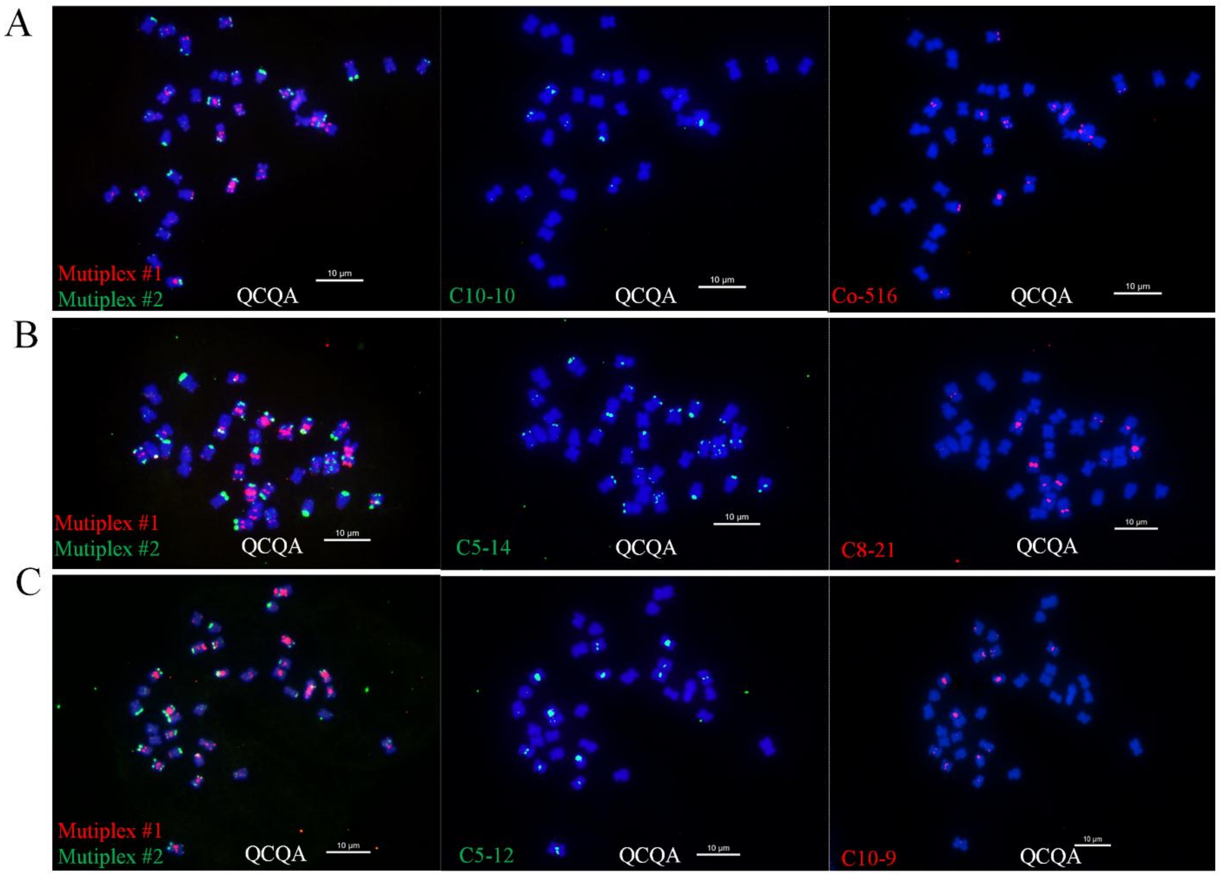

FIGURE S4 Sequential FISH localization of probes. (A) From left to right: Probe staining using Multiplex #1(red) and Multiplex #2 (green), FISH localization of probe C10-10 (green), FISH localization of probe Co-456 (red); (B) From left to right: Probe staining using Multiplex #1(red) and Multiplex #2 (green), FISH localization of probe C5-14 (green), FISH localization of probe C8-21 (red); (C) From left to right: Probe staining using Multiplex #1(red) and Multiplex #2 (green), FISH localization of probe C5-12 (green), FISH localization of probe C10-9 (red).

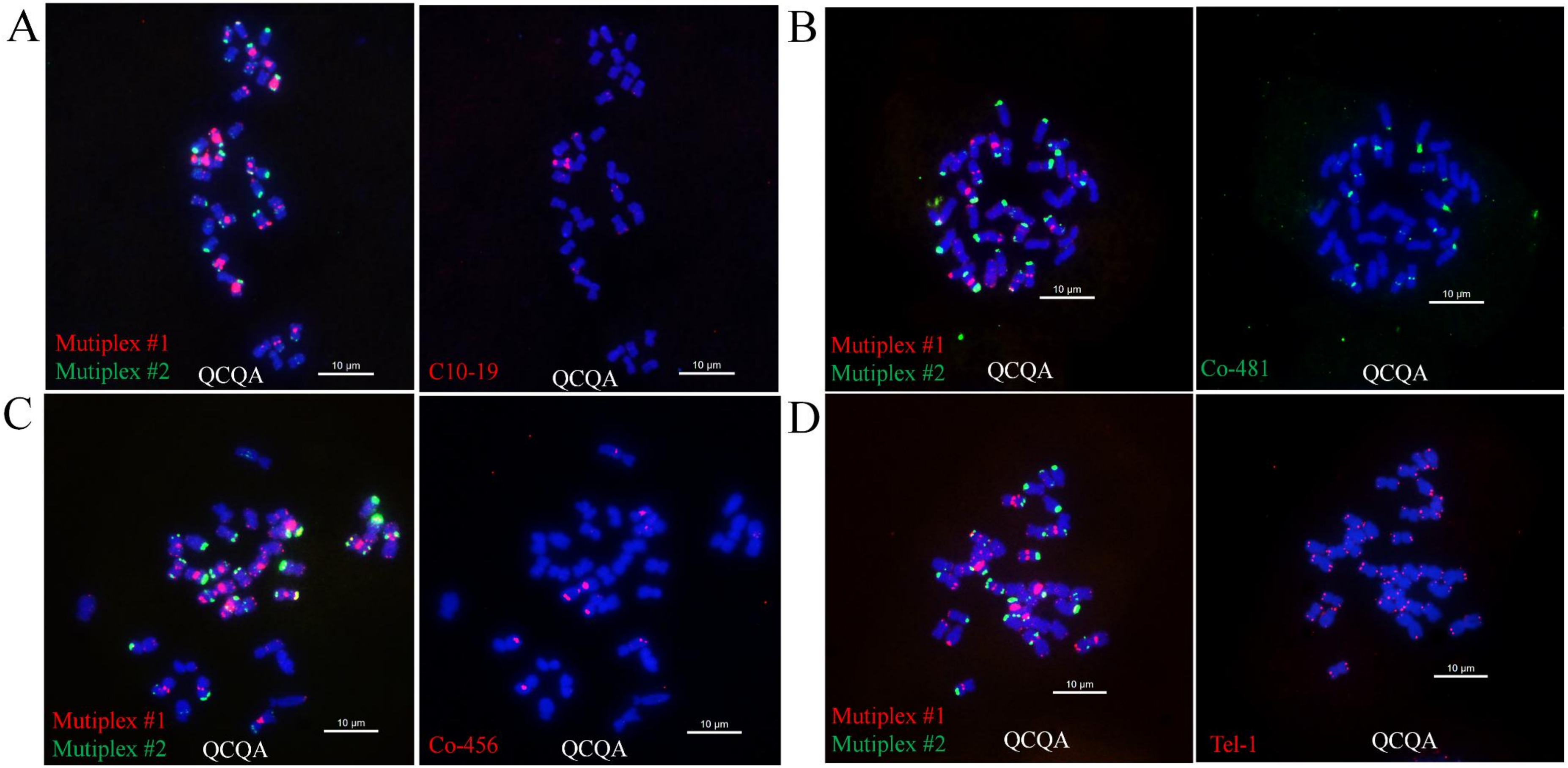

FIGURE S5 Sequential FISH localization of probe C10-19 (A), Co-481 (B), Co-456 (C), Tel-1 (D). The left column is probe staining using Multiplex #1(red) and Multiplex #2 (green), The left column is FISH localization of probes.

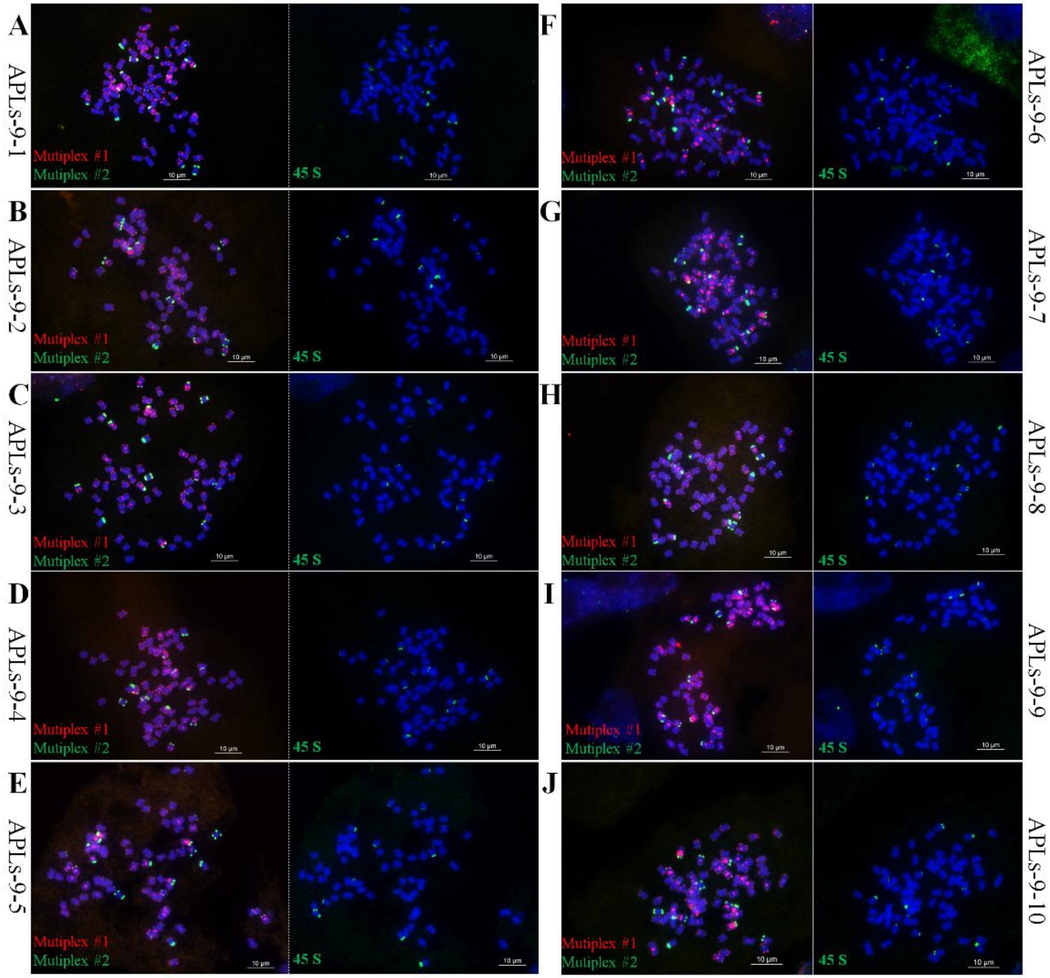

FIGURE S6 Probe staining (Left column) using Multiplex #1(red) and Multiplex #2 (green) and sequential FISH (Right column) using 45S rDNA probes (green) in ten asexual reproduction lines of octoploid *A. argyi*.

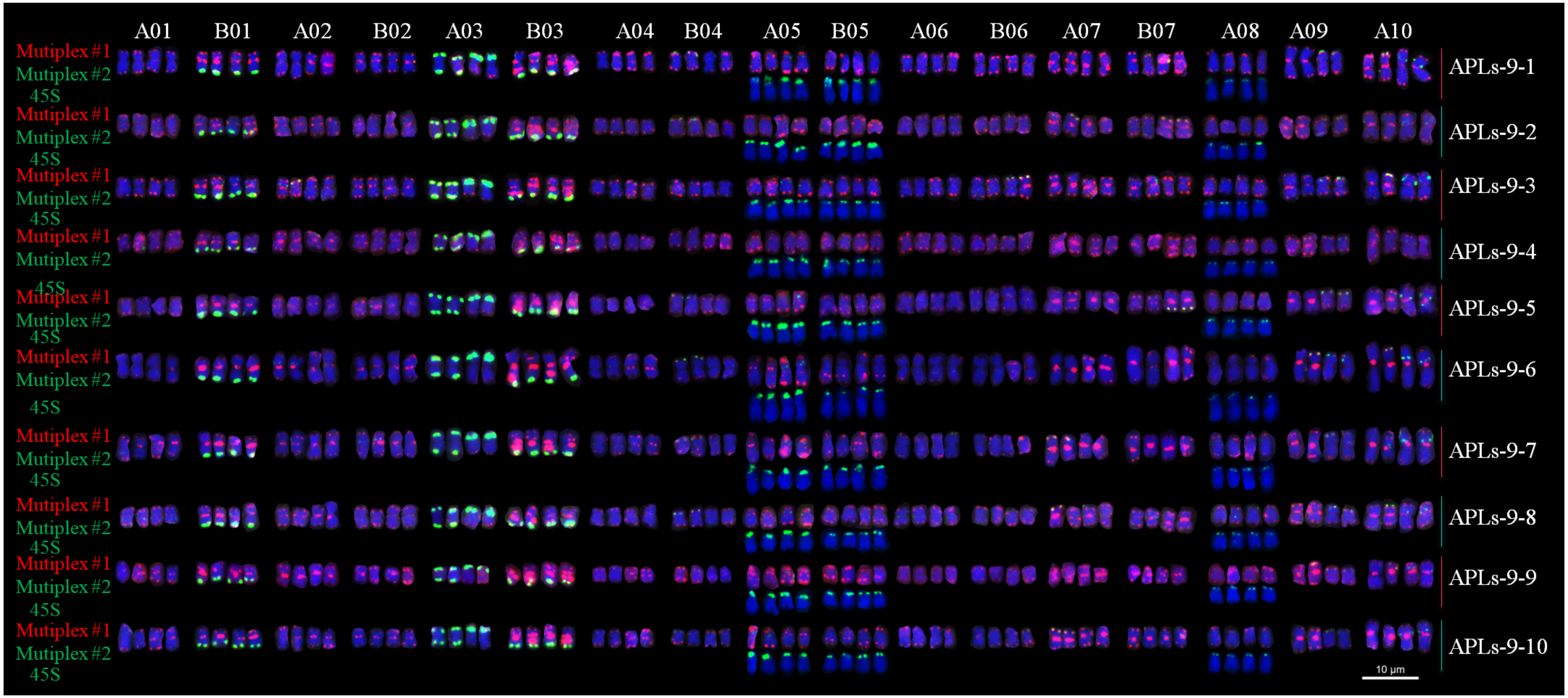

FIGURE S7 Karyotypes of ten asexual reproduction lines of octoploid *A. argyi*. The color of the probe signals is consistent with the font color.
